# The priming factor CAPS1 regulates dense-core vesicle acidification by interacting with rabconnectin3β/WDR7 in neuroendocrine cells

**DOI:** 10.1074/jbc.RA119.007504

**Published:** 2019-04-19

**Authors:** Ellen Crummy, Muralidharan Mani, John C. Thellman, Thomas F. J. Martin

**Affiliations:** From the Department of Biochemistry, University of Wisconsin, Madison, Wisconsin 53706

**Keywords:** protein-protein interaction, cellular regulation, intracellular trafficking, organellar pH homeostasis, H^+^-ATPase, CAPS, dense-core vesicle, exocytosis, rabconnectin3, WDR7

## Abstract

Vacuolar-type H^+^-ATPases (V-ATPases) contribute to pH regulation and play key roles in secretory and endocytic pathways. Dense-core vesicles (DCVs) in neuroendocrine cells are maintained at an acidic pH, which is part of the electrochemical driving force for neurotransmitter loading and is required for hormonal propeptide processing. Genetic loss of CAPS1 (aka calcium-dependent activator protein for secretion, CADPS), a vesicle-bound priming factor required for DCV exocytosis, dissipates the pH gradient across DCV membranes and reduces neurotransmitter loading. However, the basis for CAPS1 binding to DCVs and for its regulation of vesicle pH has not been determined. Here, MS analysis of CAPS1 immunoprecipitates from brain membrane fractions revealed that CAPS1 associates with a rabconnectin3 (Rbcn3) complex comprising Dmx-like 2 (DMXL2) and WD repeat domain 7 (WDR7) proteins. Using immunofluorescence microscopy, we found that Rbcn3α/DMXL2 and Rbcn3β/WDR7 colocalize with CAPS1 on DCVs in human neuroendocrine (BON) cells. The shRNA-mediated knockdown of Rbcn3β/WDR7 redistributed CAPS1 from DCVs to the cytosol, indicating that Rbcn3β/WDR7 is essential for optimal DCV localization of CAPS1. Moreover, cell-free experiments revealed direct binding of CAPS1 to Rbcn3β/WDR7, and cell assays indicated that Rbcn3β/WDR7 recruits soluble CAPS1 to membranes. As anticipated by the reported association of Rbcn3 with V-ATPase, we found that knocking down CAPS1, Rbcn3α, or Rbcn3β in neuroendocrine cells impaired rates of DCV reacidification. These findings reveal a basis for CAPS1 binding to DCVs and for CAPS1 regulation of V-ATPase activity via Rbcn3β/WDR7 interactions.

## Introduction

V-ATPases^2^ play an important role in secretory and endocytic pathways. Membrane compartments progressively acidify from the endoplasmic reticulum to Golgi to post-Golgi vesicles and from early to late endosomes (1, 2). Within the secretory pathway of neuroendocrine cells, newly formed dense-core vesicles (DCVs) further acidify due to the increased density or activity of proton-pumping V-ATPases that participate in DCV biogenesis and in cargo condensation within nascent DCVs (3–7). Decreased DCV luminal pH is crucial for the vesicular monoamine transporter (VMAT)-mediated transport of neurotransmitters into DCVs (8–10) and for the activation of prohormone-processing enzymes that catalyze conversion to mature peptide hormones (6, 11).

V-ATPase transports protons across the membrane via its membrane-spanning V0 complex that is coupled to ATP hydrolysis by an associated V1 complex (12). Several mechanisms have been proposed for the regulation of V-ATPase activity, including the reversible association/dissociation of V1 from V0. A regulator of V-ATPase of vacuoles and endosomes (RAVE) protein complex containing Rav1 protein was identified in *Saccharomyces cerevisiae* that controls V1–V0 associations (13, 14). In mammalian cells, ARF6/ARNO (15, 16) and the TORC1 complex (17) have been implicated in endosomal and lysosomal V-ATPase regulation. A screen for V-ATPase V1B1 subunit–interacting proteins identified DMXL2, an orthologue of yeast Rav1 protein, and WDR7 (18), which regulate endosomal and vesicle pH (19–22). Earlier work characterized DMXL2 and WDR7 as subunits of a rabconnectin3α/β (Rbcn3α/β) complex from a crude rat brain synaptic vesicle fraction that coimmunoprecipitated with Rab3-GEF and Rab3-GAP (23, 24), but the relationship of this complex to Rab3, a GTPase localized to DCVs, has not been determined.

The secretion of neuropeptides and biogenic amine transmitters by DCV exocytosis in neurons and endocrine cells is a tightly regulated, multistep process triggered by calcium rises. The fusion of vesicles with the plasma membrane is catalyzed by soluble *N*-ethylmaleimide–sensitive factor–associated protein receptor (SNARE) protein complexes. In neuroendocrine cells, SNARE complex formation prior to membrane fusion occurs in a vesicle priming step that requires the regulatory factors Munc18–1, ubMunc13–2, and CAPS1 (25, 26). CAPS1 was discovered as a soluble, neuronal protein that reconstitutes calcium-triggered DCV exocytosis in permeable neuroendocrine cells (27). Biochemical, genetic, and cellular studies indicate that CAPS1 plays an essential role in docking and priming DCVs at the plasma membrane prior to SNARE-mediated fusion (26, 28–33). CAPS1 has several functional domains required for its role as a priming factor, including a C2 domain that mediates CAPS1 dimerization into an active membrane-bound form (34), a pleckstrin homology (PH) domain that mediates CAPS1 binding to phosphatidylinositol 4,5-bisphosphate required for priming DCVs (31, 36, 37), and a DUF1041 + MHD1 (DAMH) domain that mediates CAPS1 binding to SNARE proteins for assembly of complexes in DCV priming (37–40). An orthologous CAPS2 (aka CADPS2) protein with high sequence similarity and similar domain architecture to CAPS1 has been characterized for a role in neuronal BDNF secretion (41–43).

Although CAPS1 is partly cytosolic in neuroendocrine cells, it also partitions onto DCVs via a C-terminal DCV-binding domain (44). C-terminally truncated CAPS1 fails to restore DCV docking and priming in CAPS1 knockdown PC12 cells, indicating that localization to DCVs is essential for CAPS1 function in vesicle exocytosis (45). However, molecules that mediate CAPS1 binding to DCVs had not been identified. CAPS1 also regulates several DCV activities that occur prior to docking and priming, including catecholamine loading (Refs. 46 and 47 but also see Ref. 48), chromogranin packaging (49), and DCV acidification (50). CAPS2 also promotes catecholamine loading, but this is not accompanied by vesicle acidification at steady state (47, 51). However, the mechanisms underlying these actions of CAPS proteins had not been determined. To clarify CAPS1 function on DCVs upstream of DCV priming, we characterized CAPS1-associated proteins in immunoprecipitates from rat brain synaptosome membrane fractions using MS. We identified Rbcn3β/WDR7 as a novel CAPS1-binding protein that is required for optimal DCV localization of CAPS1. In addition, CAPS1 and Rbcn3β/WDR7 as well as Rbcn3α/DMXL2 were found to be required for V-ATPase–mediated reacidification of DCVs. These studies reveal part of the basis for CAPS1 binding to DCVs and for CAPS1 regulation of V-ATPase activity in concert with Rbcn3β/WDR7.

## Results

### Identification of CAPS1-associated synaptosomal proteins

**Figure 1.**
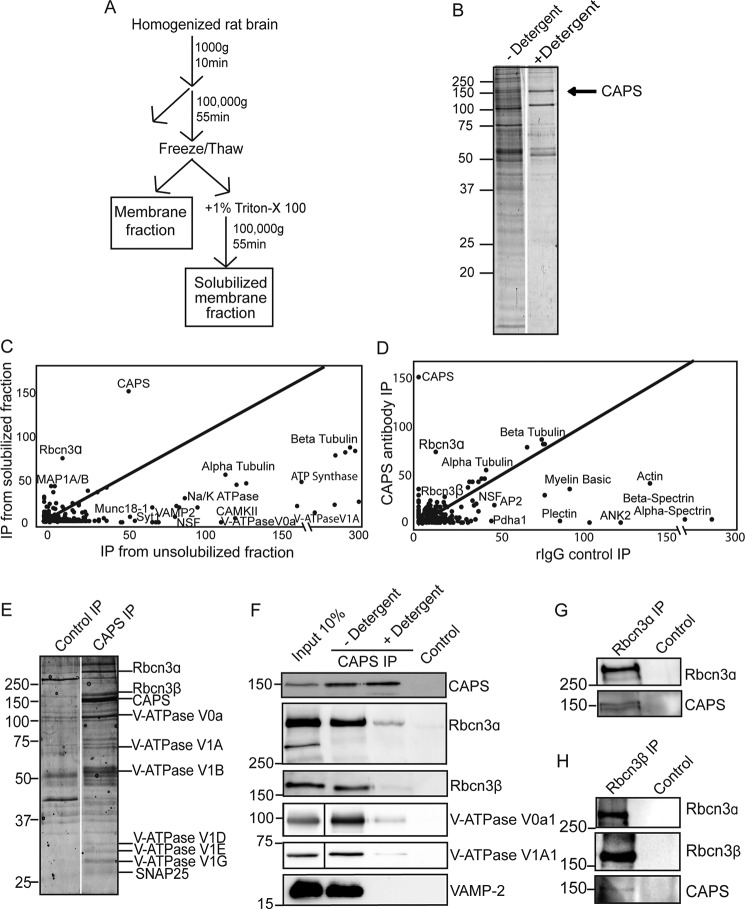
**MS analysis of CAPS immunoprecipitates from vesicle-enriched brain membrane fraction.** *A*, schematic of differential centrifugation steps to obtain crude vesicle/synaptosome preparation. *B*, representative Coomassie-stained SDS-PAGE of CAPS immunoprecipitates from membrane fraction without or with detergent solubilization. The *arrow* indicates CAPS1. *C*, scatterplot of total spectral counts of proteins coimmunoprecipitated with (*y axis*) and without (*x axis*) detergent solubilization. Proteins along the *center line* are equally abundant in both fractions, whereas proteins above the *center line* are highly enriched in CAPS1 immunoprecipitates from detergent-solubilized membranes. A subset of proteins are annotated. *D*, scatterplot of proteins coimmunoprecipitated from membrane-solubilized fraction using CAPS1 antibody (*y axis*) *versus* rabbit IgG control (*x axis*). Proteins along the *center line* are equally abundant in both fractions, whereas those enriched in CAPS1 immunoprecipitates (*IP*) are above the *center line*. A subset of proteins are annotated. *E*, Coomassie-stained proteins on SDS-PAGE from immunoprecipitates of detergent extracts with CAPS1 antibody (*right lane*) or control rabbit IgGs (*left lane*). LC-MS/MS identification of selected bands is shown. *F*, Western blot of CAPS and control immunoprecipitates confirmed LC-MS/MS identifications of Rbcn3α, Rbcn3β, and V1A1 and V0a1 domain subunits of V-ATPase. Note that vesicle proteins such as VAMP2 were de-enriched in CAPS1 immunoprecipitates of detergent-solubilized membranes. *G*, Rbcn3α antibodies (*left*) but not control IgGs (*right*) coimmunoprecipitate CAPS1. *H*, Rbcn3β antibodies (*left*) but not control IgGs (*right*) coimmunoprecipitate Rbcn3α and CAPS1. Results shown are representative of two to five independent experiments.

To detect CAPS1-associated proteins, we performed CAPS1 peptide antibody immune precipitations from membrane-enriched rat brain fractions (52). CAPS1 is an abundant protein in rat brain where 33% is membrane-associated largely in a synaptosomal fraction (27, 53). To enrich for membranes containing CAPS1, we immunoisolated membranes from lysed synaptosome fractions using a CAPS1 antibody bound to magnetic beads (Fig. 1*A*). After stringent wash steps, membrane proteins were eluted with the CAPS1 21-mer peptide immunogen, and eluates were subjected to LC-MS/MS analysis. Vesicle proteins such as V-ATPase subunits, synaptotagmin1, and VAMP2 were highly abundant in the immunoisolated membranes, consistent with a vesicle localization for CAPS1 (Fig. 1, *B* and *C*). Many additional proteins were identified in these fractions as expected for the numerous membrane proteins that co-reside on CAPS1-containing membranes.

To detect proteins that may directly associate with CAPS1, membranes were detergent-solubilized prior to CAPS1 antibody precipitation (Fig. 1, *A* and *B*). Fewer proteins were identified in these immunoprecipitates, and many vesicle-resident proteins such as VAMP2 (see Fig. 1*F*) were absent, indicating efficient detergent solubilization (Fig. 1, *B* and *C*). Cytoskeletal proteins were abundant in immunoprecipitates of detergent-solubilized membranes based on spectral counting but were disregarded because they were also highly abundant in controls using nonimmune rabbit IgGs (Fig. 1, *D* and *E*). Importantly, the most abundant noncytoskeletal proteins identified in CAPS1 immunoprecipitates from detergent-solubilized membranes consisted of Rbcn3 subunits (Fig. 1*E*). Rbcn3α (aka DMXL2) and Rbcn3β (aka WDR7) were similarly abundant and enriched in four similar independent studies. Rbcn3α and Rbcn3β were previously reported to bind each other (24, 54), and each has previously been implicated in the regulation of V-ATPase activity (18–22). Indeed, V-ATPase subunits were also enriched in CAPS1 immunoprecipitates of detergent-solubilized synaptosomes compared with controls (Fig. 1*E*).

To further investigate a potential CAPS1/Rbcn3α/Rbcn3β complex, we resolved eluates of CAPS1 immunoprecipitates by SDS-PAGE and excised Coomassie-stained bands that were visible in the CAPS1 immunoprecipitates but not in controls for LC-MS/MS analysis (Fig. 1*E*). Bands corresponding to Rbcn3α and Rbcn3β were identified, and their stoichiometry relative to CAPS1 was calculated to be 1:6 for each subunit. Bands corresponding to V-ATPase subunits were also identified but were of lesser abundance (Fig. 1*E*). To confirm LC-MS/MS identifications, we conducted CAPS1 immunoprecipitations and analyzed eluates by Western blotting with appropriate antibodies. Rbcn3α and Rabcn3β in addition to several V-ATPase subunits from both V0 and V1 complexes were present in CAPS1 but not control immunoprecipitates, consistent with the MS results (Fig. 1*F*).

To further assess CAPS1 and Rbcn3 interactions, we performed immunoprecipitations using Rbcn3α or Rbcn3β antibody to determine whether CAPS1 was pulled down with the Rbcn3 complex. CAPS1 was detected in Rbcn3α immunoprecipitates but was absent in controls (Fig. 1*G*). Rbcn3β immunoprecipitates also contained CAPS1 as well as Rbcn3α (Fig. 1*H*). Collectively, the data indicate a reproducible coenrichment of CAPS1 with the Rbcn3 complex. It appears that a substantial fraction of total CAPS1 associates with Rbcn3, whereas a smaller fraction of total Rbcn3 is associated with CAPS1 (Fig. 1, *E–H*). Because V-ATPase subunits were also enriched in CAPS1 immunoprecipitates relative to control and because the Rbcn3 complex and V-ATPase were reported to interact (18, 19, 55), we suggest that CAPS1 is part of a Rbcn3/V-ATPase complex that could regulate DCV function.

### Knockdown of Rbcn3β disrupts CAPS1 localization to DCVs

**Figure 2.**
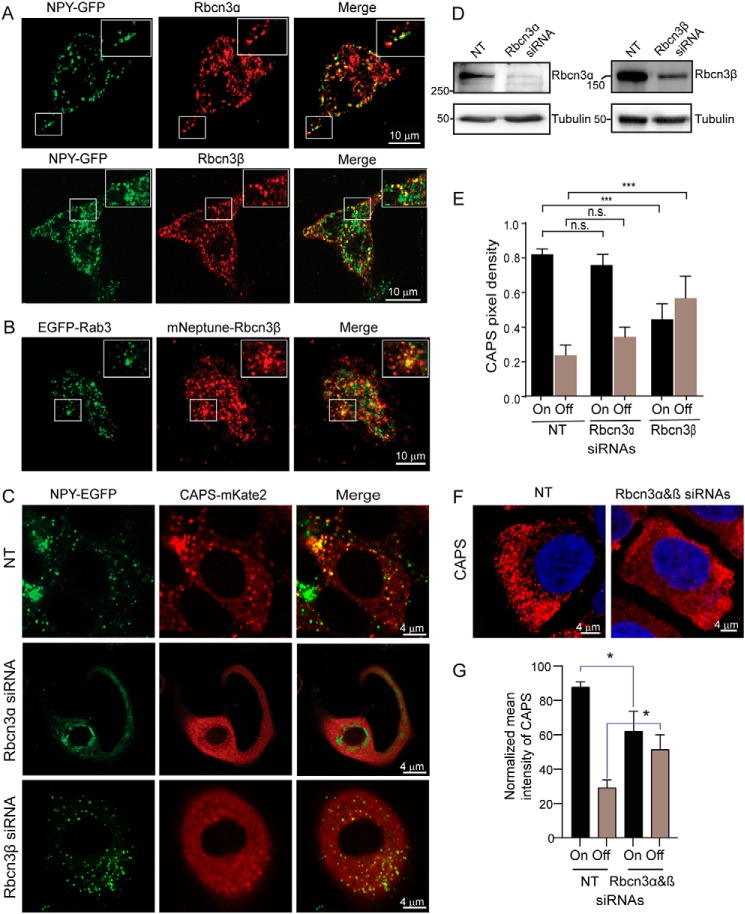
**Rbcn3β knockdown disrupts CAPS1 localization to DCVs.** *A*, confocal images of BON cells stably expressing NPY-GFP as DCV cargo that were fixed for antibody staining for Rbcn3α (*top*) or Rbcn3β (*bottom*). *Inset boxes* show enlargements. Representative images from three experiments are shown. Colocalization of Rbcn3α and Rbcn3β with NPY-GFP–containing DCVs based on Pearson correlation coefficient was 0.67 ± 0.09 (*n* = 8) for Rbcn3α and 0.52 ± 0.02 (*n* = 8) for Rbcn3β. *B*, confocal image of BON cells expressing EGFP-Rab3 and mNeptune-Rbcn3β. *Inset boxes* show enlargements. Representative images from three experiments are shown. Colocalization based on Pearson correlation coefficient was 0.53 ± 0.07 (*n* = 4). *C*, representative confocal images of BON cells stably expressing NPY-GFP and CAPS1-mKate2 and transfected with NT, Rbcn3α, or Rbcn3β siRNA pools. Pearson correlation coefficients for NPY-GFP and CAPS1-mKate2 localization were 0.73 ± 0.01 for cells transfected with NT siRNA pools, 0.58 ± 0.04 for cells transfected with siRNA pools targeting Rbcn3α, and 0.35 ± 0.03 for cells transfected with siRNA pools targeting Rbcn3β (values are the mean ± S.E. of five different cells from three independent experiments). Comparison of NT with Rbcn3β knockdown was highly significant at *p* < 0.0005. *D*, siRNA pools reduced Rbcn3α by 85% and reduced Rbcn3β by 70% as normalized to tubulin. *E*, quantification of CAPS1 on DCVs (*On*) and cytosolic CAPS1 (*Off*) for cells transfected with NT, Rbcn3α, or Rbcn3β siRNA pools. Values shown represent mean ± S.E. of five different cells from three independent experiments. ***, *p* < 0.0005. *F*, representative images of CAPS1 immunofluorescence in cells treated with nontargeting siRNA pools or a combination of individual siRNAs that target Rbcn3α and Rbcn3β. *G*, quantification of CAPS1 on membrane (*On*) and cytosolic CAPS1 (*Off*). Values shown are mean ± S.E. (*n* = 3; *, *p* < 0.05). *n.s.*, not significant.

The previous results indicated that CAPS1 binds the Rbcn3 complex in brain homogenates, but whether the Rbcn3 complex resides on DCVs and is responsible for CAPS1 localization to DCVs was unclear. To determine whether Rbcn3α and Rbcn3β are present on DCVs, we performed immunofluorescence studies in BON cells, a human pancreatic neuroendocrine cell line containing numerous large DCVs (56, 57). Cells that stably express a fluorescent DCV cargo protein, neuropeptide Y (NPY)-GFP, were used to localize endogenous Rbcn3α and Rbcn3β with specific antibodies. Both Rbcn3α and Rbcn3β were found to localize in part to NPY-GFP–containing DCVs (Fig. 2*A* and legend). Expressed mNeptune-Rbcn3β also colocalized in part with an expressed DCV-resident EGFP-Rab3 (Fig. 2*B* and legend). The results were consistent with the reported localization of Rbcn3α to DCVs in hippocampal and hypothalamic neurons (58). Localization of the Rbcn3 complex to DCVs suggests it could play a role in localizing CAPS1 to DCVs.

To determine whether the Rbcn3 complex is required for CAPS1 localization to DCVs, we knocked down Rbcn3α and Rbcn3β using siRNAs in BON cells stably expressing CAPS1-mKate and NPY-GFP. siRNA treatments reduced Rbcn3α and Rbcn3β proteins by 85 and 70%, respectively (Fig. 2*D*). With a nontargeting (NT) siRNA, 86 ± 3% of CAPS1-mKate2 localized with NPY-GFP–containing DCVs (Fig. 2, *C* and *E*), exhibiting an object-based Pearson coefficient of 0.73. Upon Rbcn3α knockdown, CAPS1-mKate2 exhibited only a trend to delocalize from DCVs (Fig. 2, *C* and *E*) with a small reduction in the Pearson coefficient to 0.58. By contrast, Rbcn3β knockdown caused a large, highly significant reduction in CAPS1 localization on DCVs to 57 ± 10% (Fig. 2, *C* and *E*) with a significant reduction in the Pearson coefficient to 0.35. This represented a dramatic redistribution of CAPS1-mKate2 from DCVs to the cytosol. That this redistribution was partial may reflect the incompleteness of the Rbcn3β knockdown (70%) or the participation of other proteins that mediate CAPS1 binding to DCVs. We also monitored endogenous immunoreactive CAPS1 (Fig. 2*F*) following the combined knockdown of Rbcn3α and Rbcn3β. The combined knockdown caused a shift of CAPS1 on membrane to the cytosol similar to that of Rbcn3β knockdown (Fig. 2*G*). Overall, the results indicate that Rbcn3β is required for the optimal localization of CAPS1 to DCVs, whereas Rbcn3α played a minor role.

**Figure 3.**
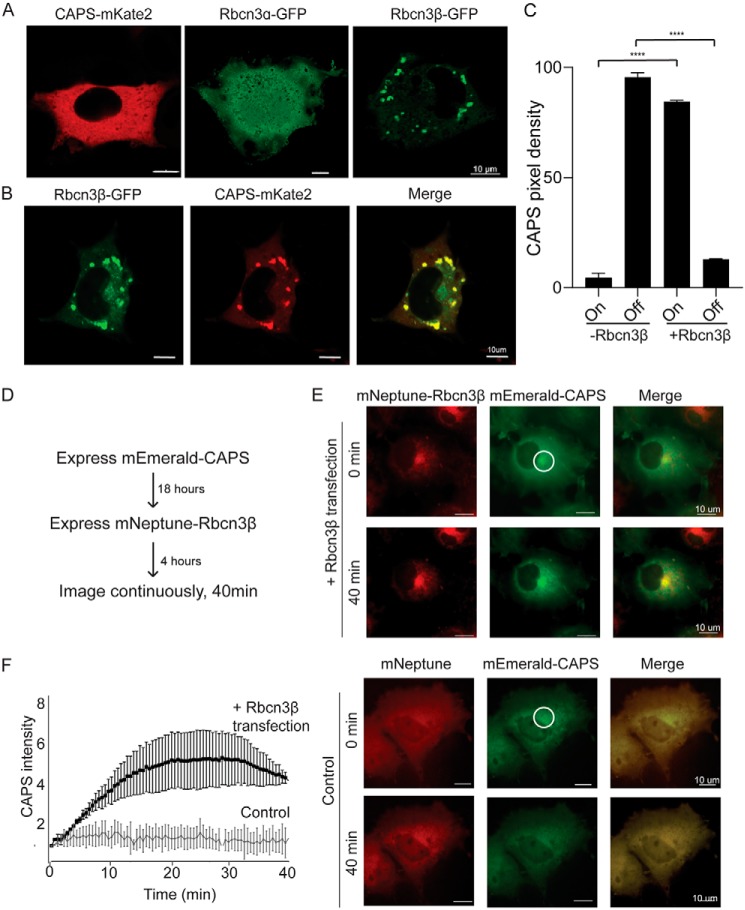
**Rbcn3β expression in COS cells recruits CAPS1 to membrane structures.** *A*, representative epifluorescence images of COS cells expressing CAPS1-mKate2, Rbcn3α-GFP, or Rbcn3β-GFP. *B*, representative confocal image of COS cells coexpressing Rbcn3β-GFP and CAPS1-mKate2 showing that CAPS1-mKate2 is recruited to Rbcn3β-containing membranes. *C*, studies were quantified by determining mean pixel intensity of CAPS1-mKate2 on Rbcn3β-GFP^+^ structures (*On*) or in cytosol (*Off*). Values represent means ± S.E. of triplicate cells in five independent experiments (****, *p* < 0.00005). *D*, sequential transfection scheme for investigating the recruitment of mEmerald-CAPS to mNeptune-Rbcn3β membrane structures. *E*, *upper panels*, epifluorescence images of mEmerald-CAPS–expressing COS cell (*middle panels*) after 4 h of mNeptune-Rbcn3β expression (*upper panels*) and 40 min later (*lower panels*). *Lower panels*, similar study to that shown in *upper panels* but with mNeptune expression. *F*, to quantify the ongoing recruitment of mEmerald-CAPS to mNeptune-Rbcn3β membranes, fluorescence was quantified within the indicated region of interest in *E* over 40 min for mNeptune-Rbcn3β– and mNeptune-expressing cells with 0-min background values subtracted. Values shown represent means ± S.E. of three independent studies (*n* = 3).

To test whether Rbcn3β positively regulates CAPS1 associations with membranes, we expressed the proteins in COS-7 cells, which lack DCVs. CAPS1-mKate2 was distributed as a soluble cytoplasmic protein in COS-7 cells (Fig. 3*A*, *left*). Rbcn3α-GFP was distributed as a soluble protein (Fig. 3*A*, *middle*), although fluorescence in the nucleus may indicate degradation and nuclear entry of GFP. By contrast, Rbcn3β-GFP was membrane-associated and localized to perinuclear vesicular structures that were likely endosomes (Fig. 3*A*, *right*). However, when CAPS1-mKate2 was coexpressed with Rbcn3β-GFP, the majority of CAPS1-mKate2 was redistributed onto Rbcn3β-positive membrane structures (Fig. 3, *B* and *C*). To image the recruitment of cytosolic CAPS1 to Rbcn3β-positive membranes, we first expressed mEmerald-CAPS1 as a cytosolic protein and subsequently expressed mNeptune-Rbcn3β (Fig. 3*D*). Time-lapse images during a subsequent 40-min period showed that cytosolic mEmerald-CAPS1 awaited Rbcn3β expression to be progressively recruited to the perinuclear Rbcn3β-positive membranes (Fig. 3, *E* and *F*). Collectively, these data indicate that CAPS1 localization to DCVs is partly dependent upon Rbcn3β and that Rbcn3β can recruit CAPS1 to membrane.

### CAPS1 directly interacts with Rbcn3β/WDR7

**Figure 4.**
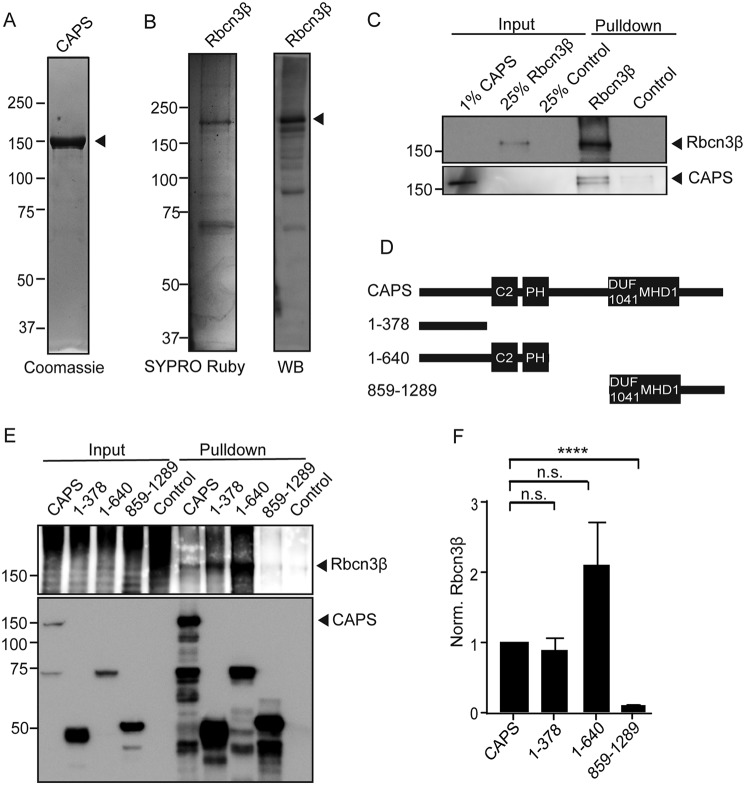
**CAPS1 directly binds to Rbcn3β.** *A*, Coomassie-stained SDS-PAGE showing CAPS1-TwinStrep purification from HEK cells. The *arrow* points to CAPS1-TwinStrep. *B*, SYPRO Ruby–stained SDS-PAGE showing Rbcn3β-GFP purification from HEK cells (*left*) and a Western blot (*WB*) of purified Rbcn3β-GFP (*right*) with Rbcn3β antibodies to show that several degradation bands were present. The *arrow* points to full-length Rbcn3β-GFP. *C*, Western blot showing binding of purified CAPS1-TwinStrep interacting with purified Rbcn3β-GFP. A representative of four similar studies is shown. *D*, scheme depicting CAPS1 domains and the HA-tagged CAPS1 fragments used for HA immunoprecipitations. C2, PH, DUF1041, and MHD domains are depicted. *E*, representative Western blot depicting pulldown of CAPS1-HA fragments and coimmunoprecipitation of Rbcn3β. 5% of the input fraction was loaded. In the concentrated input lanes for Rbcn3β, aggregation and degradation were reproducibly observed. *F*, relative Rbcn3β bound to CAPS1 fragments normalized to the amount of CAPS1 pulled down. Values shown represent means ± S.E. (*n* = 3). ****, *p* < 0.001; *n.s.*, nonsignificant.

The preceding data indicate that Rbcn3β recruits CAPS1 to membrane, but whether CAPS1 and Rbcn3β interact directly or through an intermediate protein was unclear. To address this, we expressed and purified CAPS1-TwinStrep and Rbcn3β-GFP proteins from HEK cells for binding studies. CAPS1-TwinStrep was highly purified (Fig. 4*A*), whereas the Rbcn3β-GFP protein contained several bands that were identified as degradation fragments by Western blotting (Fig. 4*B*). For binding studies, purified CAPS1-TwinStrep was mixed with the Rbcn3β-GFP (or GFP as control) immobilized on GFP nanobody–containing beads. CAPS1-TwinStrep was found to bind to the immobilized Rbcn3β-GFP but not to the GFP control (Fig. 4*C*). We conclude that CAPS1 and Rbcn3β directly interact.

CAPS1 contains a number of well-studied functional domains that are essential for vesicle priming. To determine whether any of these domains mediate binding to Rbcn3β, we coexpressed HA-tagged CAPS1 fragments (Fig. 4*D*) with Rbcn3β-GFP in HEK cells to conduct HA antibody immunoprecipitations. Compared with full-length CAPS(1–1289), a C-terminal fragment corresponding to CAPS(859–1289) containing DUF1041/MHD1 domains coimmunoprecipitated with Rbcn3β poorly at levels similar to control pulldowns (Fig. 4, *E* and *F*). By contrast, a small N-terminal fragment corresponding to CAPS(1–378) coimmunoprecipitated with Rbcn3β similarly to full-length CAPS (Fig. 4, *E* and *F*). An N-terminal fragment corresponding to CAPS(1–640) containing C2 and PH domains immunoprecipitated with Rbcn3β at least as well as full-length CAPS and CAPS(1–378). These data indicate that Rbcn3β interacts with N-terminal rather than C-terminal determinants on CAPS1. CAPS(1–378) contains essential phosphorylation sites as well as coiled-coil and intrinsically disordered regions (59), whereas CAPS(1–640) also contains C2 and PH domains. Additional studies will be required to more precisely identify the Rbcn3β-binding region of CAPS and determine its functional relevance.

### CAPS1 and Rbcn3β regulate rates of DCV acidification

The Rbcn3 complex subunits DMXL2 and WDR7 have been implicated in the acidification of endosomes and vesicles (19–21), presumably through direct interactions with V-ATPase (18, 19, 55). Indeed, DMXL2 is homologous to the yeast Rav1 protein of the RAVE complex that is required for V1–V0 V-ATPase association (18). Recent studies have shown that the knockdown of CAPS1 increases the pH of dendritic DCVs in hippocampal neurons (50). The direct interaction between CAPS1 and Rbcn3β/WDR7 shown above suggests a mechanism by which CAPS1 may regulate DCV acidification. Thus, we investigated the pH of DCVs in CAPS1, Rbcn3α, and Rbcn3β knockdown cells.

**Figure 5.**
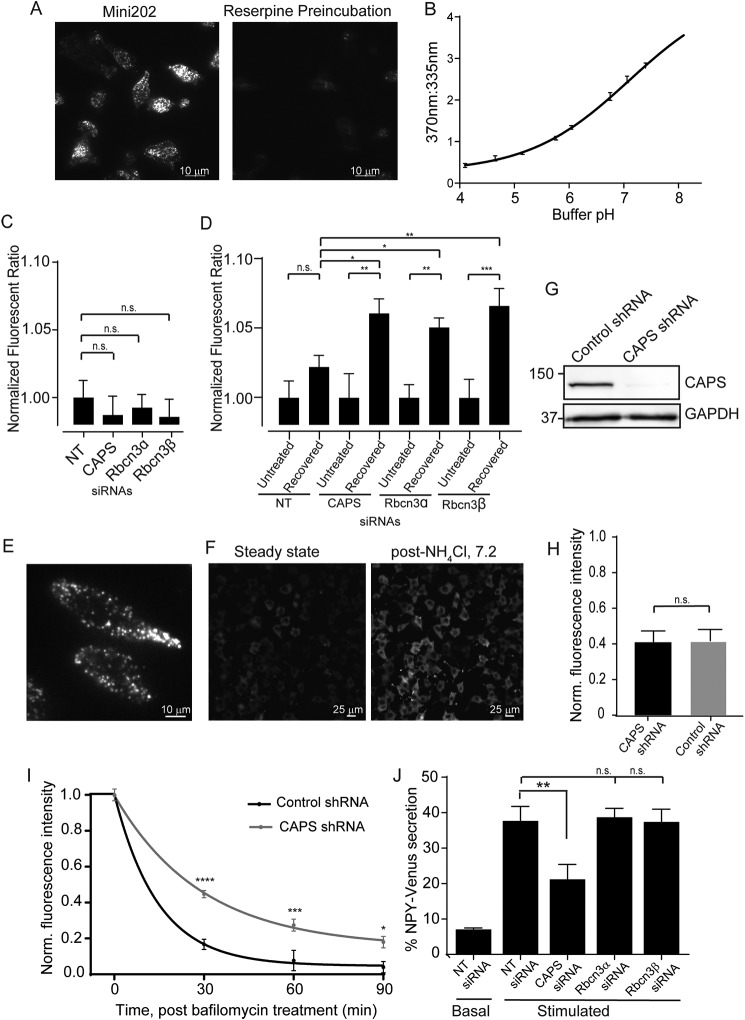
**CAPS and Rbcn3β knockdown cause acidification defects.** *A*, TIRF image at 405-nm excitation of PC12 cells loaded with compound FFN202 (*left*). FFN202 loading is decreased if cells are preincubated with 1 μm reserpine, a VMAT blocker (*right*). *B*, the 370:355 nm emission ratio of FFN202 increases with increasing buffer pH. Values shown represent means ± S.E. (*n* = 3) read from a plate reader. *C*, 370:355 nm fluorescence ratio of FFN202-loaded PC12 cells transfected with CAPS1, Rbcn3α, or Rbcn3β siRNA pools normalized to determinations with a nontargeting siRNA pool. Values are the mean ± S.E. (*n* = 3, 10 replicates each), and differences were nonsignificant (*n.s.*). *D*, 370:335 ratios of PC12 cells either loaded with FFN202 (untreated) or preincubated with 100 nm bafilomycin prior to FFN202 loading (recovered). Bafilomycin-treated cells were normalized to untreated cells in each condition. Values represent the mean ± S.E. (*n* = 3, 10 replicates each). *, *p* < 0.05; **, *p* < 0.005; ***, *p* < 0.0005. *E*, TIRF image of PC12 cells expressing NPY-GFP. *F*, lower magnification epifluorescence images of PC12 cells expressing NPY-GFP before (*left*) and after (*right*) addition of 50 mm NH_4_Cl. *G*, Western blot showing CAPS1 depletion in stable lentivirus shRNA CAPS1 knockdown PC12 cells with GAPDH loading control. *H*, ratio of NPY-GFP fluorescence before and after NH_4_Cl addition in multiple (>900) control and CAPS1 knockdown cells. Mean values ± S.E. are shown for five experiments. *I*, ratio of NPY-GFP fluorescence (±NH_4_Cl) in control or CAPS1 knockdown PC12 cells over 90 min as cells recovered from 1-h treatment with 100 nm bafilomycin. Fluorescence ratios were normalized to 1.0 at zero time. Values shown are means ± S.E. for determinations in >100 cells for each condition. *, *p* < 0.05; **, *p* < 0.005; ****, *p* < 0.00005. *J*, basal and ionomycin-stimulated NPY-Venus secretion in 10 min from BON cells was determined following transfection of the indicated siRNA pools. Mean values ± S.E. are shown. **, *p* < 0.01 (*n* = 3).

To determine whether these proteins play a role in V-ATPase–mediated acidification of DCVs in neuroendocrine cells, we utilized an assay with the fluorescent false neurotransmitter FFN202 (60). FFN202 loaded into the DCVs of neuroendocrine PC12 cells detected by imaging in TIRF microscopy (Fig. 5*A*, *left*) was determined to be a specific VMAT-dependent process blocked by reserpine (Fig. 5*A*, *right*). The fluorescence intensity of FFN202 increases at 335 nm and decreases at 370 nm if it is protonated in an acidic vesicle, whereas the intensity decreases at 335 nm and increases at 370 nm in its unprotonated form. Thus, if the knockdown of a protein neutralizes DCVs, the 370:335 emission ratio increases (Fig. 5*B*). To look for defects in vesicle acidification in the absence of CAPS1 and Rbcn3 proteins, cells were transfected with siRNA for 72 h, loaded with FFN202, and analyzed on a plate reader. Under steady-state conditions, there was no significant difference in the 370:335 ratio and thus no significant difference in DCV pH for cells transfected with CAPS1, Rbcn3α, Rbcn3β, or the nontargeting control siRNAs (Fig. 5*C*).

In other cell types, the Rbcn3 complex was found to play a modulatory rather than an essential role in V-ATPase–mediated acidification (21). The regulation of acidification by Rbcn3 was evident after treating cells with bafilomycinA1, a reversible inhibitor of V-ATPase proton pumping, and allowing recovery following washout (18). Thus, we assessed whether the knockdown of CAPS1 or Rbcn3 affected the rate of reacidification upon washout after 1-h treatment with 100 nm bafilomycin. Ninety minutes after washout, cells treated with nontargeting siRNAs had recovered the pH gradient of DCVs to levels similar to that of untreated cells (Fig. 5*D*). By contrast, cells treated with CAPS1, Rbcn3α, and Rbcn3β siRNAs contained DCVs that were significantly less acidic after recovery from bafilomycin treatment, exhibiting elevated 370:335 ratios (Fig. 5*D*). Thus, the data indicate that the Rbcn3 complex and CAPS1 alter rates of DCV acidification.

We further confirmed that CAPS1 regulates DCV acidification in neuroendocrine cells using GFP as a pH-sensitive probe (61) in PC12 cells stably expressing NPY-GFP as DCV cargo (Fig. 5*E*). To normalize for amounts of NPY-GFP, 50 mm ammonium chloride was used to neutralize DCV pH, and the ratio of cellular fluorescence before and after ammonium chloride treatment was calculated on a cell-by-cell basis (Fig. 5*F*). We compared control cells with cells in which CAPS1 was stably knocked down (Fig. 5*G*). At steady state, there was no significant difference in the fluorescence intensity ratio (−NH_4_Cl:+NH_4_Cl) between CAPS1 knockdown cells and control cells (Fig. 5*H*). However, when cells treated with bafilomycin were washed and allowed to recover, there was a significant difference between control and CAPS1 knockdown cells. Control cells recovered the pH gradient in DCVs within 90 min, whereas recovery was significantly slower in CAPS1 knockdown cells (*K* for control, 0.068 ± 0.033 min^−1^; for CAPS1 knockdown, 0.034 ± 0.006 min^−1^) (Fig. 5*I*). These data reinforce the idea that CAPS1 regulates rates of DCV acidification.

### CAPS1 but not Rbcn3 is essential for regulated DCV exocytosis

Recent studies showed that dissipating the pH gradient of DCVs strongly inhibited KCl depolarization-induced peptide secretion in neuroendocrine cells (62). These observations raised the issue whether the role of CAPS1 in DCV priming was secondary to its role in stabilizing the pH of DCVs. We tested for this possibility by comparing stimulated NPY-Venus release in cells knocked down for CAPS1, Rbcn3α, and Rbcn3β (Fig. 5*J*). Only the knockdown of CAPS1, but not that of Rbcn3α or Rbcn3β, had a substantial inhibitory effect on stimulated DCV exocytosis. The lack of correlation for siRNA effects on pH and peptide secretion suggests that the activity of CAPS1 in DCV exocytosis is independent from its activity in promoting DCV acidification as reported previously (50).

## Discussion

CAPS1 is a DCV-resident protein with a key role in vesicle docking and priming and in SNARE complex formation for DCV fusion with the plasma membrane (40, 45). However, CAPS1 has also been reported to play roles that are seemingly distinct from its function in vesicle priming. The knockdown of CAPS1 in hippocampal neurons increases the pH of DCVs from 5.8 to 6.7 (50), which is likely responsible for other knockdown phenotypes such as impaired catecholamine loading (46, 47, 63) and disruption of chromogranin packaging (3, 49). These DCV activities require V-ATPase–dependent acidification to support VMAT-mediated neurotransmitter transport (8, 9) and the sorting and condensation of granule cargo such as chromogranin (3–5). However, how soluble CAPS1 binds to DCVs as well as how CAPS1 regulates the pH of DCVs had not been determined. Here, we address both questions with evidence that CAPS1 directly binds the V-ATPase regulatory factor Rbcn3β on DCVs.

CAPS1 immunoprecipitates from vesicle-rich brain membrane fractions were enriched for Rbcn3α and Rbcn3β, which are the reported subunits of a Rbcn3 complex (54). Rbcn3 was originally discovered as a complex associated with Rab3-GEF and possibly Rab3-GAP (23, 24), although a role for the Rbcn3 complex in Rab3 regulation has not been reported. In contrast, recent studies characterized Rbcn3 as a regulator of endosome and vesicle acidification (19–22) and as an interaction partner for V1 domain subunits B, H, and C of the V-ATPase complex (18, 19, 55). Although neither Rab3-GEF nor Rab3-GAP were detectably present in CAPS1 immunoprecipitates, V-ATPase subunits were enriched, consistent with the idea that CAPS1 is part of a complex comprising Rbcn3 and V-ATPase on secretory vesicles.

The knockdown of Rbcn3β in neuroendocrine cells reduced CAPS1 localization to DCVs. Conversely, the overexpression of Rbcn3β in COS-7 cells recruited CAPS from the soluble cytoplasm to endosomes where Rbcn3β localized. Taken together with the evidence that CAPS1 directly binds Rbcn3β, this strongly indicates that Rbcn3β recruits CAPS1 to DCVs likely through interactions with the N terminus of CAPS1. However, although CAPS1 and Rbcn3 localize to DCVs in neuroendocrine cells, Rbcn3 also localizes to Rab5-positive endosomal structures (data not shown). This suggests that there are additional factors that specifically localize CAPS1 to DCVs but not endosomes. This is also suggested by previous studies that identified a C-terminal DCV-binding domain in CAPS1 (34, 44, 45). Thus, we suggest that there are multiple factors on DCVs that bind and recruit CAPS1. N-terminal CAPS binding to Rbcn3β on DCVs may be complemented by C-terminal CAPS1 interactions with other factors, possibly including VAMP2, which exhibits moderate affinity for CAPS1 binding (38). Alternatively, an interaction between Rbcn3 and Rab3 or its GEF or GAP could help guide CAPS1 to the correct membrane (23).

V-ATPase is regulated in multiple ways, one of which is the reversible assembly and disassembly of soluble V1 from the membrane-bound V0 domain (14, 64). In yeast, the Rbcn3α/DMXL2 homologue Rav1p in conjunction with Skp1p and Rav2p forms the RAVE complex that stabilizes the V1/V0 interaction to positively regulate V-ATPase proton pumping (65). Zebrafish, *Drosophila*, and mammalian cells display vesicle and endosomal acidification defects in the absence of Rbcn3α and Rbcn3β (19–21), which suggests that the Rbcn3 complex plays a V-ATPase–stabilizing role similar to the yeast RAVE complex, although this remains to be demonstrated experimentally. Our studies support the idea that Rbcn3 positively regulates acidification of DCVs and suggest that CAPS1, as part of a complex with Rbcn3, is an additional regulator.

Whether Rbcn3 serves a modulatory or essential role for endosome or vesicle acidification is controversial. The knockdown of Rbcn3α and Rbcn3β in breast cancer cells or keratinocytes dissipated the pH gradient of acidic intracellular compartments under steady-state conditions (20). By contrast, kidney cells displayed acidification deficiencies only in recovering from bafilomycin treatment (18). There were similar differences for CAPS1 knockdown between our studies and a previous study that showed decreased acidification of dendritic DCVs in primary hippocampal neurons at steady state measured with a DCV cargo protein, BDNF-EGFP (50). Our studies detected significant differences in rates of DCV acidification for CAPS and Rbcn3 knockdown in PC12 cells recovering from bafilomycin treatment. These apparent differences for acidification of DCVs may be due to differences in the composition of DCVs in different cell types. For example, the four mammalian V0a isoforms are expressed differentially on DCVs dependent on cell type (63, 66, 67). This could affect affinities for the Rbcn3 complex to influence V-ATPase regulation based on the example in yeast where the RAVE complex affects acidification of vacuoles but not Golgi cisternae because of selectivity for a vacuole-resident V0a isoform, Vph1p (68). Alternatively, there may be differences between V-ATPase levels on DCVs in primary neurons and in immortalized PC12 cells (69). Lastly, increased pH buffering of DCVs (by chromogranin) might enable rate differences in acidification but not net differences in pH at steady state to be detected after bafilomycin treatment.

How does the role of CAPS1 in acidifying DCVs relate to its function as a priming factor for vesicle exocytosis? Bafilomycin treatment does not affect DCV exocytosis in neuroendocrine cells (50, 62), and we found that Rbcn3 knockdown also fails to affect DCV exocytosis. This suggests that luminal pH *per se* does not regulate priming. It is likely that CAPS1 functions in DCV acidification at earlier steps of the regulated secretory pathway involving the biogenesis, maturation, and transport of DCVs. It has also been suggested that the assembly state of V-ATPase influences the priming and docking steps of vesicles. The V1 domain was reported to dissociate from V0 once vesicles were fully acidified and filled with neurotransmitters and that V1 dissociation could provide vesicles with better access to vesicle docking and priming factors (62, 70). Could DCV-bound V1/Rbcn3β complexes sequester CAPS1 from its priming role, potentially allowing vesicles to fully load transmitter before CAPS1 facilitates docking and priming? CAPS1 is known to dissociate from DCVs at the time of Ca^2+^-triggered exocytosis (45, 71), and it will be important to determine whether Rbcn3β or V1 subunits also dissociate from DCVs at exocytosis. In conclusion, our data show that the vesicle priming factor CAPS1 binds the V-ATPase regulatory protein Rbcn3β on DCVs and that these two factors cofunction to control rates of DCV acidification possibly by stabilizing V1–V0 V-ATPase associations.

## Experimental procedures

### Antibodies

Antibodies for Western blotting were as follows: CAPS antibody raised against the full-length protein in rabbit, purified by protein A–agarose chromatography, and used at 1:1,000; GAPDH antibody purchased from Life Technologies (catalogue number AM4300) and used at 1:4,000; Rbcn3α antibody purchased from AbCam (catalogue number ab234771) and used at 1:000; Rbcn3β antibody purchased from Sigma-Aldrich (catalogue number HPA042074) and used at 1:1000; V-ATPase V0A antibody purchased from Sigma-Aldrich (catalogue number HPA022144) and used at 1:1000; V-ATPase V1A antibody purchased from AbCam (catalogue number 137574) and used at 1:1000; GFP antibody purchased from Sigma-Aldrich (catalogue number G1544) and used at 1:2000; and HA antibody purchased from Sigma-Aldrich (catalogue number H3663) and used at 1:2000. Rbcn3β antibody for immunofluorescence was purchased from Protein Tech (catalogue number 24431-1-AP) and used at 1:50, and Rbcn3α antibody for immunofluorescence was obtained from Sigma-Aldrich (HPA039375) and used at 1:50.

### Plasmids

pLVX-NPY-GFP is described elsewhere (57). pLVX-CAPS-mKate2 lentiviral vector was cloned by removing the TfR-mCherry insert from Clontech lentiviral pLVX vector (catalogue number 63250) and inserting CAPS-mKate2 (45) with XhoI/XbaI. Rbcn3α-GFP was cloned from cDNA (catalogue number MHS6278-213246219, Dharmacon/GE Healthcare) using Gibson Assembly Master Mix (catalogue number E2611S, New England Biolabs) into pcDNA3.1 with forward primer ACGGTATGCGCCACCATGCTCTGCATCAGGTC and reverse primer AGATTCTCTGCGAATTCTAGAATGTCAAGAATTCTGTTAG, which overlaps with Rbcn3α, and forward primer GAATTCGCAGAGAATCTCTACTTCCAATC and reverse primer GGTGGCGCATACCGTCGA, which overlaps with pcDNA3.1. Rbcn3β-GFP was cloned by excising Rbcn3β from Rbcn3β-Myc-DDK (Origene, catalogue number RC213213) with HindIII and NotI and ligating into a pcDNA3.1GFP vector (Addgene, plasmid number 13031). mEmerald-CAPS was cloned by excising CAPS from CAPS-mKate2 plasmid with EcoRI and BamHI and cloning into mEmerald C1 vector (Addgene, plasmid number 53975). mNeptune-Rbcn3β was cloned by amplifying Rbcn3β from Rbcn3β-Myc-DDK by PCR using forward primer GATCTCGAGCTCAAGCTTCGGAATTCATGGCAGGAAAC and reverse primer CGCGGTACCGTCGACTGCAGTTAGACCATGAAGCGGTG and inserting into mNeptune 2.0 vector (Addgene, plasmid number 54836) using HiFi assembly (New England Biolabs, catalogue number E2621S). EGFP-Rab3A was produced using a gBlock corresponding to the rat Rab3A. Using forward primer GCAGTCGACATGGCCTCA and reverse primer CTAGGATCCTCAGCAGGC, it was inserted into pEGFP_N1 (Clontech) with SalI/BamHI. CAPS-FLAG-TwinStrep was cloned by the tobacco etch virus protease site Twin-Strep-tag (IBA-GmbH) sequence synthesized as a Gblock (see below) by Integrated DNA Technologies, Inc. into CAPS-1-myc6xHis pcDNA3.1 using EcoRI and BamHI, replacing the myc6xHis tag. pEGFP C1 was purchased from Clontech. CAPS-HA was cloned by removing CAPS from pcDNA3.1-CAPS-mKate2 (45) and inserting it into pEGFP_N1 (Clontech) with XhoI/BamHI. The original linker was replaced with a GS linker by cleaving the construct with EcoRI and using inverse PCR with forward primer GGTTCTGGCCACCACACTGGACT and reverse primer TCCTCCACCGTCATC CTCCTCGT. The EGFP tag was removed and replaced with an HA tag using reverse PCR with forward primer TTCCAGATTACGCTTAAAGCGGCCGCGACTCTAGA and reverse primer CATCGTATGGGTAGGTGGCGACCGGTGGAT. CAPS-HA fragments were cloned from full-length CAPS-HA construct using inverse PCR. The primer sequences are as follows: for CAPS(1–378)-HA: forward, GGTGGAGGAGGTTCTGG; reverse, GTCAATGATGGACGCGTTG; for CAPS(1–640)-HA: forward, GGTGGAGGAGGTTCTGG; reverse, GCCCTTGGCGTTGAG; for CAPS(859–1289)-HA: forward, GAAAATGTAGGCCGCTTAATCAC; reverse: ATGGTGGCGATACCG; and for pCMV-HA: forward, GGTGGAGGAGGTTCTGG; reverse, CATGGTGGCGATACCG.

### Cell culture

BON cells, obtained as a gift from C. M. Townsend (University of Texas Medical Branch, Galveston, TX), were cultured in 1:1 DMEM/F12 (1:1; catalogue numbers 11965-092 and 11765-062, Life Technologies) supplemented with 10% FBS (catalogue number FBS-500US, Phoenix Research) at 37 °C with 5% CO_2_. HEK293FT cells were cultured in DMEM supplemented with 10% FBS (catalogue number 16000044, Life Technologies) at 37 °C with 5% CO_2_. PC-12 cells were cultured in DMEM (catalogue number D5523, Sigma-Aldrich) supplemented with 5% horse serum and 5% calf serum (catalogue numbers SH30072.03 and 30074.03, Hyclone Laboratories) at 37 °C with 10% CO_2_. COS cells were cultured in DMEM (catalogue number 16000044, Life Technologies) supplemented with 10% FBS at 37 °C with 5% CO_2_.

### Rat brain fractionation

Animal procedures were reviewed and approved by the University of Wisconsin-Madison Institutional Animal Care and Use Committee. Outbred Harlan Sprague-Dawley rats were euthanized using CO_2_ inhalation followed by decapitation. Tissue samples were dissected and put into sucrose buffer (20 mm HEPES, 2 mm EDTA, 2 mm EGTA, 1 mm DTT, 320 mm sucrose, protease inhibitors (catalogue number 11873580001, Roche Applied Science)). Tissue was homogenized with 10 strokes in a Potter-Elvehjem homogenizer. Sample was spun at 1000 × *g* for 10 min. Supernatant was harvested and spun at 17,000 × *g* for 55 min to obtain a crude synaptosomal fraction. The pellet was resuspended in sucrose buffer, aliquoted, and flash frozen. Prior to immunoprecipitation, sample was thawed and diluted into PBS (1.8 mm KH_2_PO_4_, 10 mm Na_2_HPO_4_, 2.7 mm KCl, 137 mm NaCl, pH 7.4) in the presence of protease inhibitors for nonsolubilized synaptosomal fraction or solubilized in 1% Triton X-100, incubated on ice for 20 min, and spun at 100,000 × *g* for 1 h.

### CAPS antibody immunoprecipitations

Protein A Dynabeads (catalogue number 1001D, Thermo Fischer Scientific) washed two times with PBS were incubated with a rabbit polyclonal CAPS peptide antibody (59) at room temperature for 30 min. After washing unbound antibody with PBS, beads were incubated in brain homogenate for 1 h at 4 °C with agitation. Beads were washed three times for 5 min in PBS with or without 1% Triton X-100 as indicated. After the final wash, beads were put into a new tube, and CAPS peptide (SEKEKEELERLQKEEEERKKR) diluted in PBS was added to elute CAPS and interacting proteins. After 30 min of elution, supernatant was harvested for SDS-PAGE or LC-MS/MS analysis.

### Mass spectrometry

Protein eluates from CAPS antibody or control IgG beads were precipitated by adding 10% TCA, 10% acetone, and spinning at 16,000 × *g* for 15 min. Pellets were washed with cold acetone twice and then methanol with 10-min spins between washes. Pellets were air-dried for 10 min, and sample was reduced in 2 m urea, 1 mm DTT, 10% methanol, 50 mm ammonium bicarbonate and incubated at 50 °C for 15 min. 1 mm iodoacetamide was added, and sample was incubated for 15 min in the dark. Alkylation was quenched with additional DTT to 2 mm. Samples were digested in trypsin and incubated at 37 °C overnight. Reactions were quenched with TFA. Samples were cleaned up with C_18_ tips (catalogue number 87782, Thermo Fischer Scientific). Peptides were analyzed by nano-LC-MS/MS using the Agilent 1100 nanoflow system (Agilent) connected to a hybrid linear ion trap–orbitrap mass spectrometer (LTQ-Orbitrap XL, Thermo Fisher Scientific) equipped with an EASY-Spray^TM^ electrospray source. Chromatography of peptides prior to mass spectral analysis was accomplished using a capillary emitter column (PepMap® C_18_, 3 μm, 100 Å, 150 × 0.075 mm; Thermo Fisher Scientific) onto which 6 μl (of 30 μl total) of extracted peptides was automatically loaded. The nano-HPLC system delivered solvents A (0.1% (v/v) formic acid) and B (99.9% (v/v) acetonitrile, 0.1% (v/v) formic acid) at 0.60 μl/min to load the peptides (over a 45-min period) and 0.3 μl/min to elute peptides directly into the nanoelectrospray with a gradual gradient from 0% (v/v) B to 40% (v/v) B over 195 min and concluded with a 5-min fast gradient from 40% (v/v) B to 100% (v/v) B at which time a 1-min flash-out at 100% (v/v) B took place. As peptides eluted from the HPLC column/electrospray source, survey MS scans were acquired in the Orbitrap with a resolution of 100,000, and up to five of the most intense peptides per scan were fragmented and detected in the ion trap over the 300–2000 *m*/*z*; redundancy was limited by dynamic exclusion. Raw MS/MS data were converted to mgf file format using MSConvert (ProteoWizard: Open Source Software for Rapid Proteomics Tools Development). Resulting mgf files were used to search against the UniProt *Rattus norvegicus* amino acid database (September 18, 2013 download) with decoy reverse entries and common lab contaminants included (54,780 total entries) using in-house Mascot search engine 2.2.07 (Matrix Science) with variable methionine oxidation and asparagine/glutamine deamidation. Peptide mass tolerance was set at 20 ppm, and fragment mass was set at 0.8 Da. Protein annotations, significance of identification, and spectrally based quantification was done with help of Scaffold software (version 4.3.2, Proteome Software Inc., Portland, OR). Protein identifications were accepted if they could be established at greater than 95.0% probability and contained at least two identified peptides. Protein probabilities were assigned by the Protein Prophet algorithm (35). Proteins that contained similar peptides and could not be differentiated based on MS/MS analysis alone were grouped to satisfy the principles of parsimony. For in-gel digests, peptides were analyzed by nano-LC-MS/MS using the Agilent 1100 nanoflow system connected to a hybrid linear ion trap–orbitrap mass spectrometer (LTQ-Orbitrap Elite^TM^, Thermo Fisher Scientific) equipped with an EASY-Spray electrospray source using methods similar to those described above.

### shRNA and siRNA knockdown

Lentivirus and stable cell lines were generated as described in Zhang *et al.* (57). Lentiviruses expressing the CAPS1 target sequence AATCCGTCTTGCTGAACTAGT or the DMXL2 target sequence AAAGATGCAATCGAGGTATGT were used to transduce PC12 cells in the presence of 10 μg/ml protamine sulfate. Lentiviruses expressing a nontargeting sequence CAACAAGATGAAGAGCACCAA were used as a control. Cells were transfected for siRNA knockdown with Magnetofection SilenceMag (catalogue number SM10200, OZ Biosciences) or with RNAiMAX (catalogue number 13778030, Invitrogen) with 50 nm siRNA (Dharmacon). After 48–72 h, live cells were imaged on a confocal microscope (A1R+, Nikon) equipped with GaAsP detectors and an oil objective (60×/NA1.40; Plan Apo), or cells were harvested for Western blotting for target validation. To target DMXL2 in BON cells, the siRNA pool consisted of GGGCAUAAAUUCUCAUAAA, CAUCUUAGCUCCCACAGUA, GAACAUUGGCUACAGGUUA, and CGACAUCUCUCUCGAACUA. To target WDR7 in BON cells, the siRNA pool consisted of GAAGCAAGCUACCGCUAUU, GCACUGUUGUUUGGUCAUA, GAGCACCGCUUCAUGGUCU, and GAUCGAUGCUUGGAGGUGA. To target CAPS1 in PC12 cells, the siRNA pool consisted of GACUAUAACUUGUGCAAUG, CAAUAUGCACCAUGUUUAA, UAGACGAACUAAUUGAAGA, and CUUAAUCACUCCCGCCAAA. To target WDR7 in PC12 cells, the siRNA pool consisted of GGAUAGAGAGUUGGUAAUU, UCUCAAUGCUUGUGACGAA, GGCAAGGGCUAAAGGCGAA, and GAAAGCUACUGCAGGCGAA. To target DMXL2 in PC12 cells, the siRNA pool consisted of UAAUAAACCUGUCCCAGUA, CAUCGAAUCCUAAACCUUA, GCUAUUCUCUCCCAUUUAG, and UGUCAAAUUUGGAGACGUU. The negative control siRNA contains at least four mismatches to any human or rat sequence consisting of UAAGGCUAUGAAGAGAUAC.

### Fluorescence microscopy imaging and image analysis

Cells were plated on poly-d-lysine–coated coverslips and transfected with Lipofectamine 2000 reagent (Life Technologies) according to the manufacturer's recommendation. Cells were fixed in 4% formaldehyde (8 min) and permeabilized with 0.1% Triton X-100 in PBS with 5% BSA (10 min). After blocking with 5% BSA in PBS, primary antibodies were incubated overnight at 4 °C and stained with secondary antibodies for 1 h. Coverslips were mounted with SlowFade reagent (Life Technologies). Samples were imaged on a confocal microscope (A1R+, Nikon) equipped with GaAsP detectors and an oil objective (60×/NA1.40; Plan Apo) or on a STORM/TIRF microscope (Nikon) equipped with a laser (MLC400B, Agilent) charge-coupled device camera (iXon Ultra, Andor) and an oil objective (100×/NA1.49; ApoTIRF). The microscopes were controlled by Nikon Elements software. Image acquisition was performed at ambient temperature, and images were analyzed with ImageJ (National Institutes of Health) or Nikon Elements software.

### CAPS binding to Rbcn3β

For CAPS-TwinStrep-FLAG purification, HEK293FT cells were transfected by calcium phosphate transfection with a CAPS-TwinStrep-FLAG expression plasmid. Cells were washed with PBS; flash frozen; resuspended in Buffer W (catalogue number 2-4998-000, IBA) with 1% Triton X-100, 1 mm DTT, and protease inhibitors; and passed through a 23-gauge needle 10 times. Unbroken cells and nuclei were pelleted at 45,000 × *g* for 20 min at 4 °C. Supernatant was added to Strep-TactinXT Superflow beads (catalogue number 2-4030-002, IBA) and incubated for 1 h at 4 °C. Beads were washed five times in Buffer W, and CAPS was eluted in Buffer E (catalogue number 2-4998-000, IBA). Protein was exchanged into 25 mm Tris, pH 7.4, 150 mm NaCl using Zeba Spin Desalting Columns (catalogue number 87766, Thermo Fischer Scientific). Unused protein was flash frozen and stored at −80 °C. For Rbcn3β purification, HEK293FT cells were transfected by calcium phosphate transfection with Rbcn3β-GFP expression plasmid. Cells were washed with PBS, solubilized in Lysis Buffer (25 mm Tris, pH 7.4, 450 mm NaCl, 1% NP-40, 1 mm EDTA, 5% glycerol) in the presence of a protease inhibitor mixture for 15 min. Nuclei and unbroken cells were pelleted at 16,000 × *g*, while GFP-Trap MA beads (catalogue number gtma-10, Chromotek) were washed three times in Lysis Buffer. Lysate was added to beads and incubated 30 min on ice followed by four 10-min washes in Lysis Buffer. Purified CAPS was added to beads in binding buffer (25 mm Tris·HCl, pH 7.4, 75 mm NaCl, 0.5% NP-40, 0.5 mm EDTA, 2.5% glycerol) and incubated for 1 h. Proteins were eluted with Laemmli sample buffer and boiled for 10 min prior to SDS-PAGE.

### HA pulldowns and Western blotting

HEK293FT cells were transfected by calcium phosphate transfection. Cells were washed with PBS and solubilized in Lysis Buffer (25 mm Tris, pH 7.4, 150 mm NaCl, 1% NP-40, 1 mm EDTA, 5% glycerol) in the presence of a protease inhibitor mixture for 15 min at 4 °C. Nuclei and unbroken cells were pelleted at 16,000 × *g*, while beads were washed three times in Lysis Buffer. Lysate was incubated with Pierce Anti-HA Magnetic Beads (catalogue number 88836, Thermo Fischer Scientific) for 1 h on ice with agitation. Beads were washed with Wash Buffer (25 mm Tris·HCl, pH 7.4, 75 mm NaCl, 0.5% NP-40, 0.5 mm EDTA, 2.5% glycerol) two times, and proteins were eluted in Laemmli sample buffer and boiled for 10 min. Samples were loaded onto 8% polyacrylamide gels. After electrophoresis (Bio-Rad), proteins in the gel were electrotransferred to nitrocellulose membranes (0.45-μm pore; Bio-Rad). Membranes were blocked with 5% skim milk in PBS with 0.1% Tween 20 (PBST) for 1 h at room temperature and incubated with primary antibodies in 1% BSA in PBST overnight at 4 °C. Primary antibody was detected by incubation with horseradish peroxidase–conjugated secondary antibodies for 1 h at room temperature. Blots were developed by an enhanced chemiluminescence kit (Pierce) and imaged by an ImageQuant LAS 4000 system (GE Healthcare).

### DCV pH determinations

For plate assays with FFN202 to assess pH, PC12 cells were seeded in 96-well plates and transfected 18 h after plating with siRNA pools (Rbcn3α, M-014049-01-005; Rbcn3β, M-012867-01-0005; CAPS, M-019218-004-0005; nontargeting, D-001206-13-005, all from Dharmacon) with DharmaFECT4 (catalogue number T-2004-01, Dharmacon) according to the manufacturer's recommendation. After 72 h, cells were treated with 100 nm bafilomycinA1 (catalogue number B1793, Sigma-Aldrich) or 1% DMSO for 30 min. Cells were washed, incubated with 20 μm FFN202 (ab120867, AbCam) for 1 h, and washed five times for reading at 335 and 370 nm using Tecan Infinite M1000 PRO. The 370:335 ratio was calculated for each well. Outliers were removed using the ROUT method, and statistical significance was calculated using one-way analysis of variance with Prism 6 software. DCV pH values could not be obtained by calibration because of the lack of a suitable microscope with 335- and 370-nm excitation. For additional microscopy assays of DCV pH, PC12 cells expressing NPY-EGFP were seeded in poly-d-lysine–coated MatTek dishes (catalogue number P35G-1.5-14-C, MatTek Corp.). Movies were taken on a STORM/TIRF microscope (Nikon) at 20×. Ten seconds after acquisition start, ammonium chloride was added to a final concentration of 50 mm. Total cell fluorescence before and after ammonium chloride was calculated using ImageJ/Fiji software. Outliers were removed using the ROUT method, and statistical significance was calculated with analysis of variance using Prism 6 software.

### NPY-Venus secretion assay

For secretion assays, siRNAs were delivered into NPY-Venus–expressing BON cells by reverse transfection by mixing siRNA (50 nm final) with Metafectamine SI (Biontex Laboratories GmbH) or RNAiMAX (Life Technologies) reagent and transfection buffer in 96-well plate wells. Cell suspension was added and incubated for 48 h. For acute stimulation of NPY-Venus secretion, cells were washed with 200 μl of PSS-Na (145 mm NaCl, 5.6 mm KCl, 2.2 mm CaCl_2_, 0.5 mm MgCl_2_, 15 mm HEPES, 5.6 mm glucose) and incubated in 100 μl of PSS-Na/ionomycin (1.25 μm) or PSS-Na/DMSO (same concentration as ionomycin) for 10 min at 37 °C. Stimulation buffer was transferred to a black-bottom plate; cells were lysed with 100 μl of PSS-Na, 1% Triton X-100; and cell lysate was transferred to a separate black-bottom plate. The fluorescence of NPY-Venus of both stimulation buffer and cell lysates was determined in a Safire II plate reader (Tecan), and background fluorescence of buffer was subtracted. The ratio of NPY-Venus in stimulation buffer and total (stimulation buffer + cell lysate) was used to calculate percent secretion.

## Author contributions

E. C., M. M., and T. F. J. M. conceptualization; E. C., M. M., and J. C. T. data curation; E. C., M. M., and J. C. T. formal analysis; E. C., M. M., and J. C. T. investigation; E. C., M. M., and J. C. T. methodology; E. C. and T. F. J. M. writing-original draft; E. C. and T. F. J. M. writing-review and editing; T. F. J. M. funding acquisition; T. F. J. M. project administration.

## Footnotes

2 The abbreviations used are:

V-ATPase: vacuolar-type H^+^-ATPase
DCV: dense-core vesicle
CAPS: Ca^2+^-dependent activator protein for secretion
RAVE: regulator of V-ATPase of vacuoles and endosomes
GEF: guanine nucleotide exchange factor
GAP: GTPase-activating protein
PH: pleckstrin homology
Rbcn3: rabconnectin3
MHD: Munc13 homology domain
DUF1041: domain of unknown function
VMAT: vesicular monoamine transporter
DMXL2: Dmx-like 2
WDR7: WD repeat domain 7
SNARE: soluble *N*-ethylmaleimide–sensitive factor–associated protein receptor
BDNF: brain-derived neurotrophic factor
NPY: neuropeptide Y
EGFP: enhanced GFP
NT: nontargeting
HEK: human embryonic kidney
HA: hemagglutinin
TIRF: total internal reflection fluorescence
GAPDH: glyceraldehyde-3-phosphate dehydrogenase
DMEM: Dulbecco's modified Eagle's medium
FBS: fetal bovine serum
GaAsP: gallium arsenide phosphide
NA: numerical aperture
NP-40: Nonidet P-40
ROUT: robust regression and outlier removal.
